# Definitive radiotherapy for adenoid cystic carcinoma of main bronchus: a case report with 10-year follow-up

**DOI:** 10.3389/fonc.2025.1608613

**Published:** 2026-01-20

**Authors:** Sunghyun Nam, Hyungsoon Kim, Doyoon Kim, Jihoon Maeng, Hyo Sup Shim, Dae Joon Kim, EunYoung Kim, Min Hee Hong, Seo Hee Choi, Jaeho Cho

**Affiliations:** 1 Yonsei University College of Medicine, Seoul, Republic of Korea; 2 Department of Pathology, Severance Hospital, Yonsei University College of Medicine, Seoul, Republic of Korea; 3 Department of Thoracic and Cardiovascular Surgery, Yonsei University College of Medicine, Seoul, Republic of Korea; 4 Division of Pulmonary and Critical Care Medicine, Department of Internal Medicine, Severance Hospital, Yonsei University College of Medicine, Seoul, Republic of Korea; 5 Division of Medical Oncology, Department of Internal Medicine, Yonsei Cancer Center, Yonsei University College of Medicine, Seoul, Republic of Korea; 6 Department of Radiation Oncology, Yonsei Cancer Center, Heavy Ion Therapy Research Institute, Yonsei University College of Medicine, Seoul, Republic of Korea

**Keywords:** adenoid cystic carcinoma, case report, radiotherapy, intensity-modulated radiotherapy, tracheal neoplasm

## Abstract

**Introduction:**

Tracheal adenoid cystic carcinoma (ACC) is a rare malignancy with a slow but relentless course. Surgical resection is the standard treatment, but in cases where surgery is not feasible, definitive radiotherapy serves as an alternative approach.

**Case description:**

We report the case of a 55-year-old female patient diagnosed with unresectable ACC of the left main bronchus near the carina. Due to the high surgical risk of pneumonectomy with carinal reconstruction, the patient was treated with definitive intensity-modulated radiation therapy (IMRT) using a simultaneous integrated boost technique. A total dose of 69 Gy in 30 fractions was delivered. The patient tolerated the treatment well without significant acute or late complications. Follow-up imaging demonstrated a durable local response, with near-complete remission of the primary tumor. Ten years post-treatment, the patient remains free of local recurrence, with slow-progressing pulmonary metastases under surveillance.

**Conclusion:**

This case highlights the potential of definitive IMRT using a hypofractionated dose scheme in achieving long-term local control for unresectable tracheal ACC.

## 1 Introduction

Adenoid cystic carcinoma (ACC) is a rare malignant neoplasm that primarily arises in the head and neck region, accounting for approximately 1% of all malignancies in this area and 10% of all salivary gland tumors (1–3). ACC is characterized by indolent but persistent growth; however, despite an initial 5-year overall survival (OS) rate of 68% to 90%, long-term prognosis remains relatively poor, with 10-year and 15-year OS rates declining to 52% and 28%, respectively (1, 2). This poor long-term outcome is largely attributed to the tumor’s propensity for perineural invasion, local recurrence, and distant metastasis, which may occur as late as 25 years after diagnosis (1, 2, 4).

Although ACC typically originates in the salivary glands, it can also arise in the trachea and bronchi, where it develops from submucosal glands of the tracheobronchial mucosa (3, 5). Tracheal ACC, first described by Morgagni in 1762, presents a particular challenge due to its slow but relentless progression and anatomically complex location (6). To date, literature on tracheal ACC remains scarce. However, as with ACC in other locations, surgical resection, often followed by adjuvant radiotherapy (RT), is considered the primary treatment option (2, 3, 7). Recent data indicate that complete surgical resection can achieve 5-year overall survival rates of approximately 70% to 90% (8–10). The feasibility of surgery, however, is highly dependent on tumor size, location, and the patient’s pulmonary function, and radical resection is often unfeasible when critical structures such as the main bronchus or carina are involved.

In such cases, definitive RT serves as an essential alternative. However, the role of definitive RT is still unclear. It is usually offered for patients with incomplete resection or inoperable disease, with reported local control rates ranging widely from 20% to 70% depending on the technique and dose used (6, 11–15). Given the intrinsic radioresistance of ACC, achieving effective tumor control requires high-dose RT. Recent advances in modern RT techniques, including intensity-modulated radiation therapy (IMRT) and particle therapy, have significantly improved dose delivery precision while minimizing exposure to normal tissues. However, studies on definitive RT for tracheal ACC remain limited, primarily consisting of small retrospective series and case reports, which often use conventional RT techniques with suboptimal RT doses (6, 13–15). Here, we report a rare case of unresectable ACC of the main bronchus adjacent to the carina, successfully treated with IMRT as definitive therapy and achieving durable local disease control during the follow-up period of 10 years. To the best of our knowledge, this represents one of the longest reported follow-up durations for definitive IMRT in this setting. This case supports the potential of modern RT as an effective curative approach for tracheal ACC, contributing to the growing evidence supporting definitive RT in this rare malignancy.

## 2 Case description

In June 2015, a 55-year-old female patient presented with a persistent cough that had developed in January following a diagnosis of influenza. She had no history of smoking and had no other medical history except for hypertension. Physical examination revealed a patient with good performance status. Head and neck examination showed no palpable cervical lymphadenopathy or masses in the salivary glands. On chest auscultation, breath sounds were significantly diminished over the left lung field, consistent with airway obstruction, whereas the right lung field was clear. There was no evidence of digital clubbing or peripheral edema. Routine laboratory tests, including blood and urine analyses, were unremarkable.

**Figure 1:**
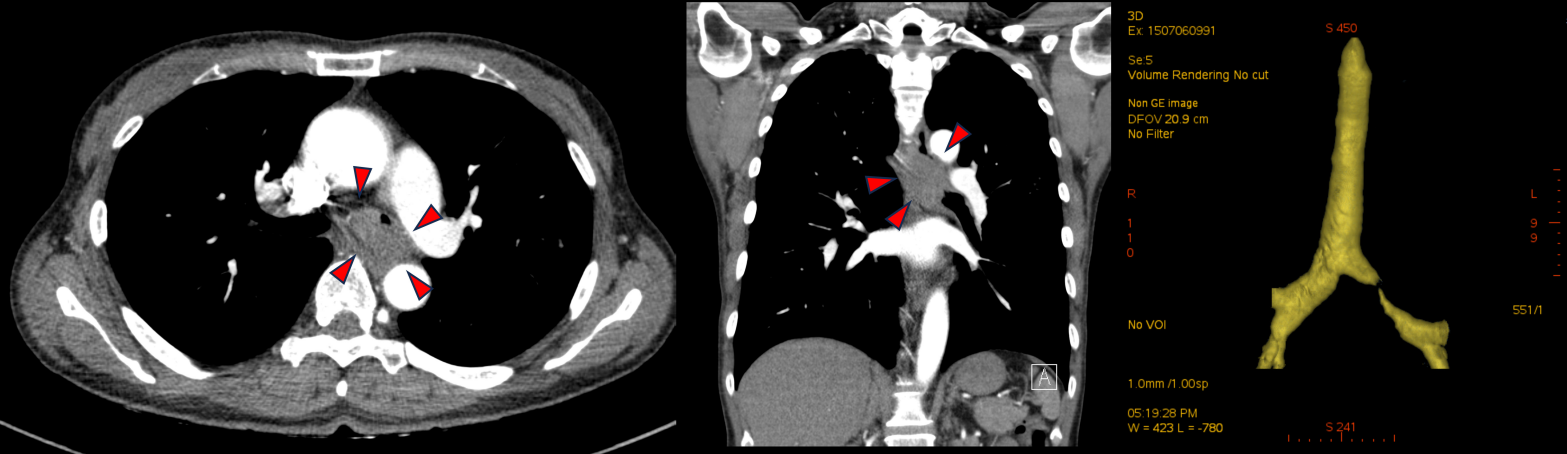
Pre-treatment chest computed tomography (CT) scan. CT scan revealed a 3.5-cm enhancing mass in the left main bronchus, causing significant luminal narrowing and impending obstruction. The mass was also abutting the thoracic aorta, raising suspicion of a possible malignancy.

**Figure 2:**
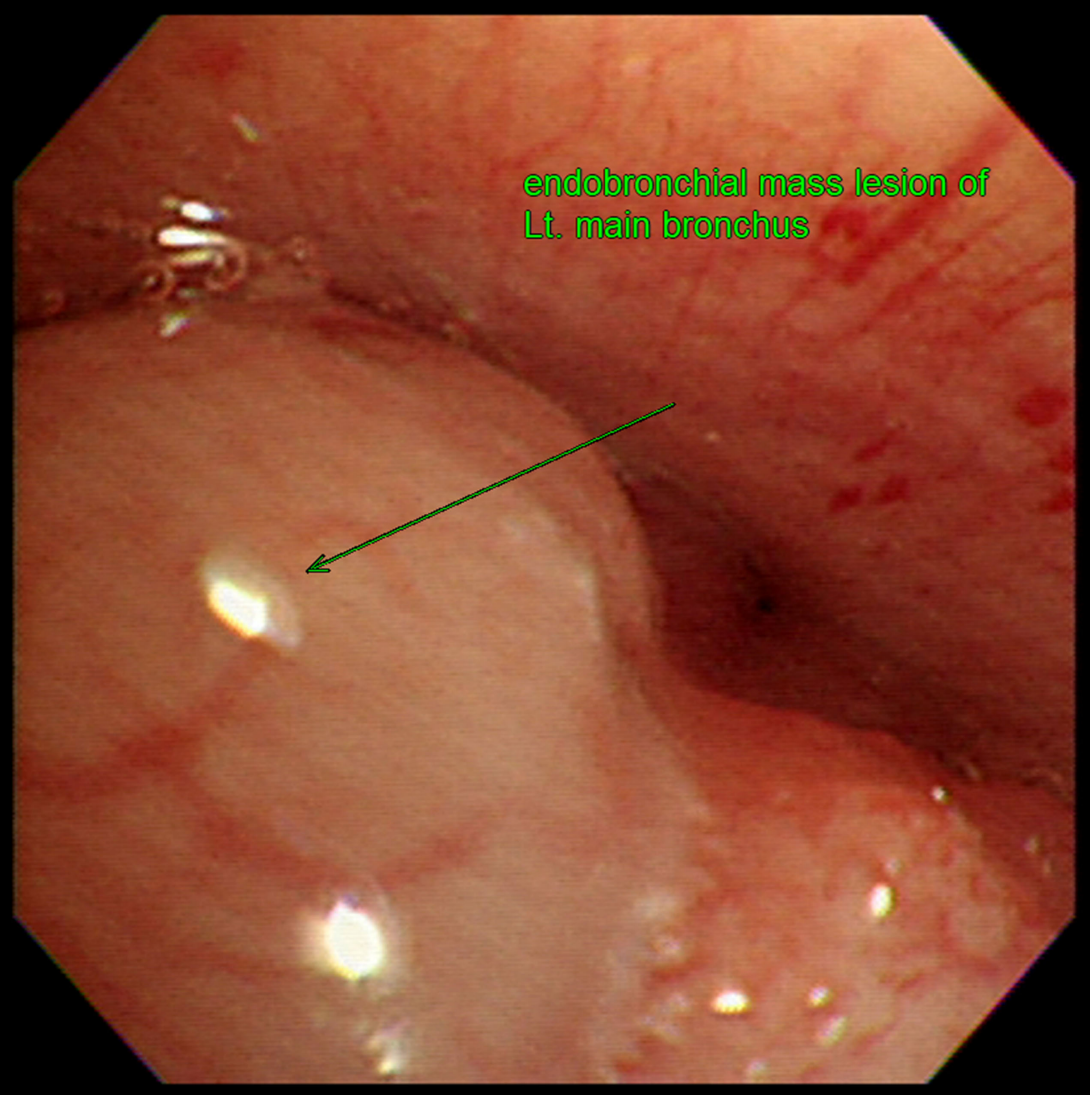
Pre-treatment fiberoptic bronchoscopy findings. Bronchoscopy revealed an endobronchial mass located 0.8 cm distal to the carina within the left main bronchus, exhibiting erythematous changes and vascular engorgement on the surface.

**Figure 3:**
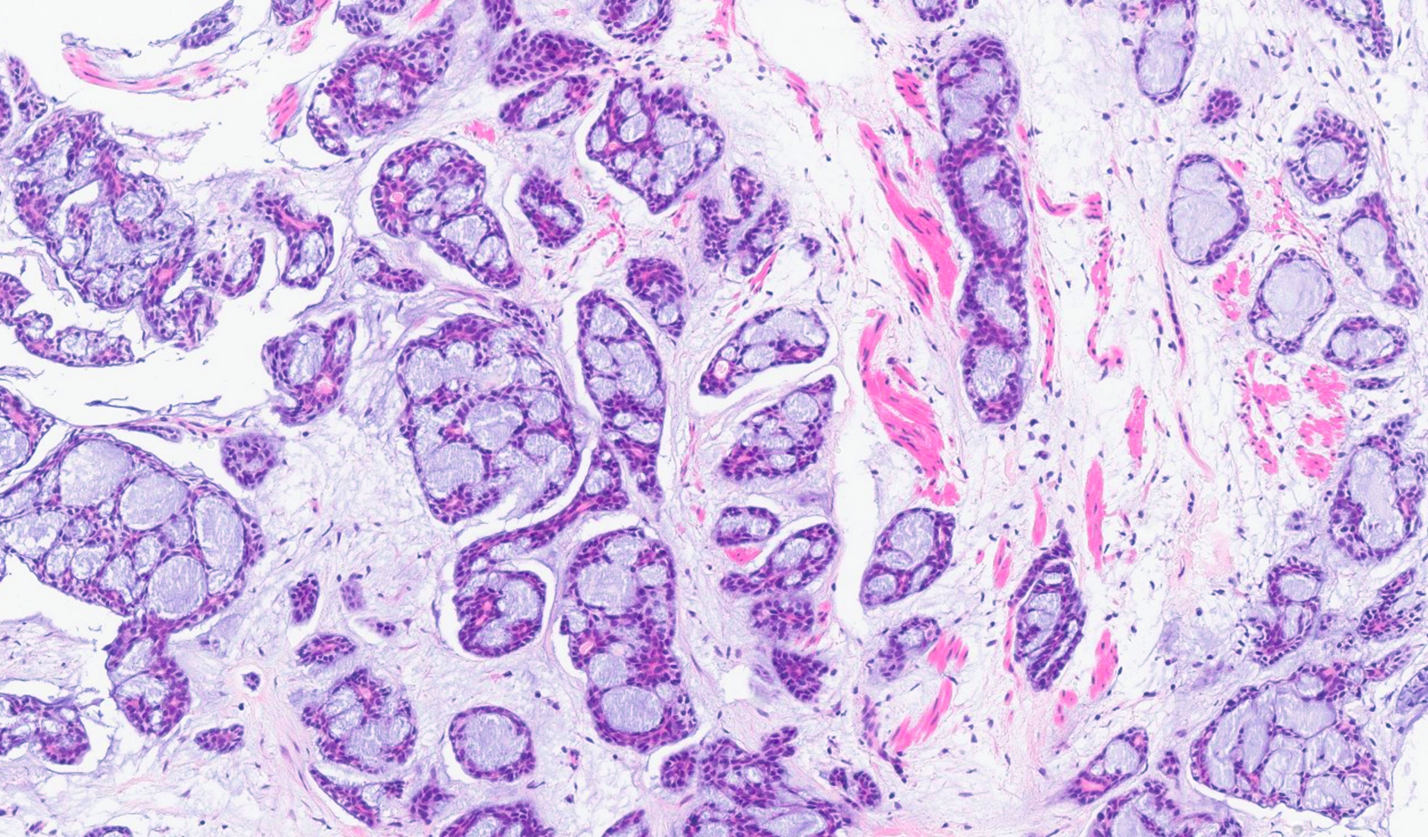
Histopathological finding of left main bronchial biopsy showed infiltration of adenoid cystic carcinoma. Cribriform growth pattern is predominant. Hematoxylin and eosin stain, ×100.

Given the persistent nature of her symptoms, a comprehensive pulmonary evaluation was performed under the initial impression of chronic bronchitis. Initial chest x-ray revealed newly appearing, numerous tiny nodules in the bilateral middle lung fields. A subsequent chest computed tomography (CT) scan demonstrated multifocal centrilobular nodules, peribronchial consolidation, and bronchiectasis in both upper lobes and the right middle lobe. Additionally, a 3.5-cm enhancing mass was identified in the left main bronchus, causing impending luminal obstruction (**Figure 1**). The mass was found to be abutting the thoracic aorta and extending toward the esophagus, causing external compression. Esophagogastroduodenoscopy was performed to evaluate possible esophageal invasion, but the esophageal mucosa was intact with no evidence of intrinsic invasion or fistula formation, despite the external compression noted on imaging. Further staging with positron emission tomography-computed tomography (PET-CT) confirmed only mild uptake of 18F-fluorodeoxyglucose (FDG) in the bronchial mass but showed no evidence of distant metastasis (**Supplementary Figure 2A**). A fiberoptic bronchoscopy with Narrow Band Imaging (NBI) revealed an endobronchial mass located 0.8 cm distal to the carina within the left main bronchus. Notably, NBI visualization identified extensive submucosal vascular engorgement involving the main carina, extending approximately 2.5 cm proximally toward the trachea and 2 cm into the right main bronchus. This finding suggested submucosal tumor spread beyond the visible gross tumor (**Figure 2**). Bronchial washing cytology was positive for malignancy, and histopathological examination of a bronchoscopic biopsy confirmed the diagnosis of ACC, showing a predominant cribriform growth pattern (**Figure 3**).

## 3 Diagnostic assessment

The tumor was staged as T3N0M0, according to the American Joint Committee on Cancer staging system. The lung perfusion scan showed pulmonary blood flow of 16.4% in the left lung and 83.6% in the right lung, suggesting reduced left lung perfusion due to narrowing of the left main bronchus (**Supplementary Figure 1**). Therefore, prompt treatment was required for the endobronchial mass causing the left bronchus narrowing.

Following diagnosis, a multidisciplinary discussion was conducted involving thoracic surgeons, radiation oncologists, medical oncologists, and radiologists to determine the optimal treatment strategy. Surgical resection was initially considered as the primary treatment option. However, the extensive submucosal spread involving the carina and right main bronchus ruled out lung-preserving techniques such as sleeve lobectomy. Radical resection would have necessitated a left pneumonectomy with carinal reconstruction under cardiopulmonary bypass. However, the patient’s pulmonary function was critically compromised, with a forced expiratory volume in 1 second (FEV1) of 0.94 L (51%). Based on the lung perfusion scan results, the predicted post-pneumonectomy FEV1 was calculated to be only 786 mL. Given the prohibitively high risk of postoperative ventilatory failure and operative mortality, and after a thorough discussion with the patient regarding these risks, the medical team and the patient jointly decided to proceed with definitive RT using IMRT.

**Figure 4:**
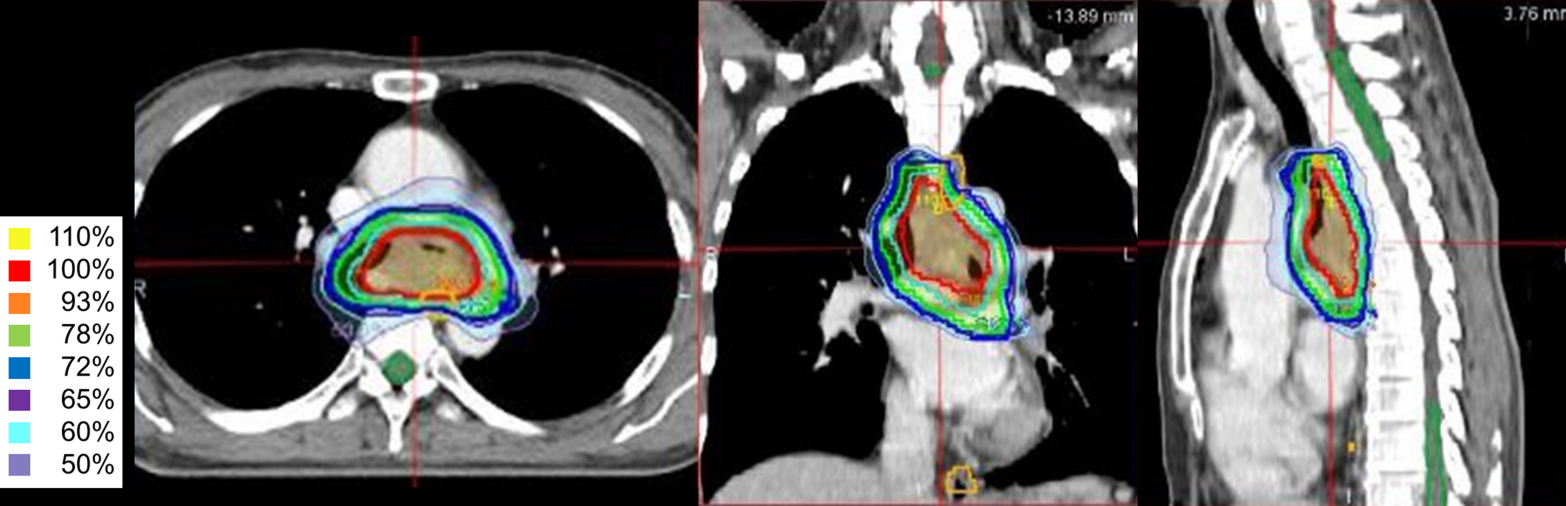
Intensity-modulated radiation therapy plan with isodose line distributions. The red contour represents planning target volume (PTV) 1, the sky blue contour represents PTV2, the green contour represents PTV3, and the blue contour represents PTV4. Using the simultaneous integrated boost (SIB) technique, the PTVs received 2.3 Gy, 2.15 Gy, 1.8 Gy, and 1.65 Gy per fraction, respectively. The total prescribed dose to the primary tumor was 69 Gy in 30 fractions, ensuring optimal dose delivery while sparing adjacent critical structures.

Definitive RT was planned using Volumetric Modulated Arc Therapy (VMAT) with 6 MV photons on the RayStation treatment planning system (RaySearch Laboratories, Stockholm, Sweden). The target volumes were delineated based on the gross tumor volume without elective nodal irradiation. Stepwise margins of approximately 3–5 mm were added to the gross tumor volume to define four planning target volumes (PTV1–4) (**Figure 4**). A simultaneous integrated boost (SIB) technique was employed, delivering 2.3 Gy (Total 69 Gy), 2.15 Gy (64.5 Gy), 1.8 Gy (54 Gy), and 1.65 Gy (49.5 Gy) per fraction to the respective PTVs. The total prescribed dose was 69 Gy in 30 fractions, equivalent to 70.7 Gy in equivalent dose in 2-Gy fractions (α/β = 10), administered five times per week from August 2015 to October 2015. Dose constraints were strictly met to minimize exposure to organs at risk. The mean lung dose was 7.84 Gy with a V20 of 7.53%, and the spinal cord D0.03cc was 22.22 Gy. For the esophagus, D0.03cc was 71.35 Gy with a V50Gy of 14.88%, which was considered acceptable given the tumor’s proximity. Treatment adherence was excellent; the patient completed the prescribed radiotherapy course without interruption. She tolerated the treatment well, with no acute toxicities exceeding Common Terminology Criteria for Adverse Events Grade 2.

A PET-CT scan performed 2 months after IMRT demonstrated a markedly decreased FDG uptake in the left main bronchial mass, indicating a favorable treatment response (**Supplementary Figure 2B**). At 5 months post-treatment, chest CT showed a significant reduction in tumor size, with only a residual 1.2-cm soft tissue infiltration, consistent with a partial response (**Supplementary Figures 2C, D**). Subsequent routine follow-up chest CT scans were performed every 3 to 6 months. The reduced bronchial mass remained stable, and notably, no severe late adverse events commonly associated with RT, such as tracheal stenosis, tracheoesophageal fistula, or hemoptysis, were observed. At 3 years and 6 months post-treatment (April 2019), chest CT and PET-CT detected multiple lung nodules in both lungs, suspected to be lung metastases. Given the slow progression and small tumor burden, the oncologist opted for close observation rather than immediate intervention. By March 2025—almost 10 years post-treatment—the patient remained under regular follow-up. Clinician-assessed outcomes indicate a preserved ECOG performance status of 0. There has been minimal progression of lung lesions, and the patient reports no respiratory symptoms. The primary tracheal lesion has remained in a near-complete response state, with no evidence of tumor regrowth, tracheal stenosis, or respiratory symptoms.

Patient Perspective Reflecting on the past 10 years, the patient expressed high satisfaction with the treatment outcome. She noted that the RT course was manageable, and she has since maintained a normal daily life without any respiratory symptoms, such as dyspnea or cough. She feels that avoiding high-risk surgery was the right choice for preserving her quality of life and continues to adhere to her surveillance schedule with a positive outlook.

## 4 Discussion

Tracheal ACC is the second most common primary tumor of the trachea, accounting for 10–20% of cases, second to squamous cell carcinoma (SCC). It typically occurs in middle-aged adults, with no clear sex predilection, and unlike SCC, smoking is not a known risk factor. The tumor most commonly arises in the lower trachea but can also occur in the mainstem or lobar bronchi, with rare cases in the segmental bronchi or extrathoracic trachea. Histologically, it is identical to ACC of the salivary glands, originating from the submucosal glands of the tracheobronchial tree. ACC often presents as a polypoid lesion but may also extend longitudinally or circumferentially along the airway. Despite being a low-grade tumor, it has a strong propensity for local recurrence and late metastasis, spreading via direct extension, submucosal or perineural invasion. Lymphatic spread is rare (<20%), whereas hematogenous metastases occur in over 50% of cases, most frequently affecting the lungs, followed by the brain, bones, liver, kidneys, skin, abdomen, and heart.

Surgical resection remains the standard treatment, offering the best chance for long-term control, especially when complete excision with negative margins is achievable (9). However, many cases pose significant surgical challenges due to tumor location near critical structures such as the carina and the tumor’s tendency for submucosal spread. Surgery may not be feasible in cases of extensive tumor spread along the airway, invasion of adjacent critical structures, or large tumor burden. This difficulty was exemplified in the present case, where NBI confirmed extensive submucosal invasion extending to the contralateral bronchus and trachea in a patient with prohibitive pulmonary function (predicted post-pneumonectomy FEV1 < 800 mL), thereby precluding curative resection. Given these limitations, RT has emerged as a crucial alternative for patients who are not surgical candidates.

Historically, RT has been used primarily as an adjuvant treatment after incomplete surgical resection or for symptom relief (16). According to long-term follow-up data from Shanghai Chest Hospital (8), among 109 patients with tracheal ACC, positive surgical margins were reported in approximately 84%, and the majority received adjuvant RT. In more recently treated patients, adjuvant RT has been more actively administered, with the 5-year and 10-year OS rates of 90.8% and 61.2%, respectively. Recommended doses range from 45–65 Gy, depending on resection margins. Thus, adjuvant RT is strongly recommended for patients with tracheal ACC, particularly for patients with residual tumors.

Although data on definitive RT are limited, several studies have suggested that this approach provides long-term disease control. A Korean long-term follow up study (11) reported an overall response rate of 77.8% for definitive RT, with the 5-year and 10-year local progression-free survival rates of 66.7% and 26.7%, respectively. Two-thirds of patients survived for more than 5 years, with tracheal stenosis occurring in only one case without tumor recurrence. Notably, patients receiving higher RT doses via brachytherapy boost had better outcomes, with no local progression at 5 years. Several studies have reported the feasibility of lower doses of external radiation therapy followed by endotracheal brachytherapy boost (17, 18). These findings support definitive RT as a viable option and suggest safe dose escalation should be considered.

Modern radiation techniques, such as IMRT and particle therapy, including proton and carbon-ion radiotherapy, have the potential to improve treatment outcomes. However, due to the rarity of tracheal ACC and the need for long-term follow-up to evaluate late toxicities, clinical data remain limited. IMRT enables precise dose delivery while sparing adjacent organs, making it particularly useful for tumors near critical structures like the lungs, esophagus, and heart. A recent study comparing conventional treatment with IMRT in postoperative patients with tracheal ACC with positive surgical margins has reported significantly improved OS and local progression-free survival, with the 5-year rates of 88.9% and 64.3%, respectively (19). Additionally, a recent case report has described definitive RT using IMRT followed by a proton boost (20). Regarding carbon-ion radiotherapy, while it has not been reported specifically for tracheal ACC, studies have demonstrated its efficacy in head and neck ACC (21–24). Theoretically, particle therapy offers superior dose conformity due to the Bragg peak effect, effectively reducing the “low-dose bath” to surrounding normal tissues. However, its application to tracheal ACC requires caution. Unlike head and neck cases, applying high-LET radiation to centrally located tumors abutting critical serial organs like the esophagus and trachea poses significant risks. Range uncertainties could lead to severe late toxicities, such as tracheoesophageal fistula or perforation. Therefore, particle therapy might not be suitable for cases with extensive invasion into luminal structures until more robust safety data are available. In this context, high-precision IMRT currently remains a reliable and safe curative modality.

Although this study is limited by being a single case report rather than a comparative series regarding surgical versus non-surgical approaches, a distinguishing feature is the almost 10-year follow-up duration. To the best of our knowledge, this is among the longest follow-up periods reported for definitive IMRT in tracheal ACC. Given the scarcity of data on definitive radiotherapy for this rare entity, this long-term outcome provides valuable insights into the durability and safety of high-dose IMRT as a curative modality. Specifically, this patient received hypofractionated IMRT, delivering 69 Gy in 30 fractions via a SIB technique. This approach ensured effective tumor control while minimizing toxicity. The patient tolerated the treatment well, with no significant acute or late complications, such as tracheal stenosis or radiation-induced fibrosis. Additionally, the successful use of dose escalation through hypofractionation supports its feasibility as an alternative to conventional fractionation. Future studies should refine the balance between hypofractionation and normal tissue tolerance.

While this case highlights the efficacy of high-dose IMRT in achieving durable local control, distant metastasis remains a concern. Despite excellent control of the primary lesion, pulmonary metastases were detected 3.5 years post-RT. This aligns with existing literature indicating the lungs are the most common site of metastases, occurring in over 50% of cases. In this case, metastases exhibited slow progression, and observation rather than immediate systemic therapy was chosen. Nevertheless, given the limited efficacy of current systemic treatments, further research is needed to explore effective therapeutic strategies (25). In addition to cytotoxic chemotherapy, advances in targeted therapy and immunotherapy may play a role in future treatment paradigms, particularly for patients with controlled primary tumors but persistent systemic disease.

In conclusion, this case underscores the potential of IMRT as a definitive treatment for unresectable tracheal ACC, demonstrating long-term local control with minimal toxicity. The use of a hypofractionated high-dose regimen contributed to the sustained response, supporting the feasibility of dose escalation strategies in ACC treatment. However, we acknowledge the limitations of this study, including its design as a single case report, the lack of molecular, and the absence of detailed pathology images beyond the primary cribriform pattern. While surgery remains the preferred approach when feasible, definitive RT provides an effective alternative, particularly for patients with inoperable disease. Further research involving multi-institutional data is warranted to optimize RT protocols, explore systemic therapy options, and establish long-term follow-up strategies to improve outcomes for patients with this rare malignancy.

## Data Availability

The original contributions presented in the study are included in the article/**Supplementary Material**. Further inquiries can be directed to the corresponding author.

## References

[1] Coca-Pelaz A Rodrigo JP Bradley PJ Vander Poorten V Triantafyllou A Hunt JL . Adenoid cystic carcinoma of the head and neck–An update. Oral Oncol. (2015) 51:652–61. doi: 10.1016/j.oraloncology.2015.04.005, PMID: 25943783

[2] Fang Y Peng Z Wang Y Gao K Liu Y Fan R . Current opinions on diagnosis and treatment of adenoid cystic carcinoma. Oral Oncol. (2022) 130:105945. doi: 10.1016/j.oraloncology.2022.105945, PMID: 35662026

[3] Mu X Li Y He L Guan H Wang J Wei Z . Prognostic nomogram for adenoid cystic carcinoma in different anatomic sites. Head Neck. (2021) 43:48–59. doi: 10.1002/hed.26443, PMID: 32864833

[4] Ladefoged C Bisgaard C Petri J . Solitary renal metastasis 23 years after extirpation of a bronchial adenoid cystic carcinoma. Scand J Thorac Cardiovasc Surg. (1984) 18:245–8. doi: 10.3109/14017438409109900, PMID: 6098964

[5] Geiger JL Ismaila N Beadle B Caudell JJ Chau N Deschler D . Management of salivary gland Malignancy: ASCO guideline. J Clin Oncol. (2021) 39:1909–41. doi: 10.1200/JCO.21.00449, PMID: 33900808

[6] Maziak DE Todd TR Keshavjee SH Winton TL Van Nostrand P Pearson FG . Adenoid cystic carcinoma of the airway: thirty-two-year experience. J Thorac Cardiovasc Surg. (1996) 112:1522–31; discussion 31-2. doi: 10.1016/S0022-5223(96)70011-9, PMID: 8975844

[7] Desai HM Thakare R Amonkar GP Karkhanis V Joshi JM . Adenoid cystic carcinoma of the trachea. Indian J Pathol Microbiol. (2015) 58:516–8. doi: 10.4103/0377-4929.168889, PMID: 26549080

[8] Yang H Yao F Tantai J Zhao Y Tan Q Zhao H . Resected tracheal adenoid cystic carcinoma: improvements in outcome at a single institution. Ann Thorac Surg. (2016) 101:294–300. doi: 10.1016/j.athoracsur.2015.06.073, PMID: 26431923

[9] Gaissert HA Grillo HC Shadmehr MB Wright CD Gokhale M Wain JC . Long-term survival after resection of primary adenoid cystic and squamous cell carcinoma of the trachea and carina. Ann Thorac Surg. (2004) 78:1889–96. doi: 10.1016/j.athoracsur.2004.05.064, PMID: 15560996

[10] Luo Y Teng J Wang Z Hong Q Zou H Li L . Clinical characteristics, treatment and prognosis of primary tracheal adenoid cystic carcinoma: A multicenter retrospective study. Cancer Med. (2025) 14:e70877. doi: 10.1002/cam4.70877, PMID: 40249221 PMC12007181

[11] Je HU Song SY Kim DK Kim YH Jeong SY Back GM . A 10-year clinical outcome of radiotherapy as an adjuvant or definitive treatment for primary tracheal adenoid cystic carcinoma. Radiat Oncol. (2017) 12:196. doi: 10.1186/s13014-017-0933-6, PMID: 29202770 PMC5716005

[12] Piorek A Tabor S Jaskiewicz P Kasprowicz A Zaborowska-Szmit M Winiarczyk K . A case of adenoid cystic carcinoma of trachea: treatment complications and radiotherapy role. J Contemp Brachytherapy. (2021) 13:588–92. doi: 10.5114/jcb.2021.109853, PMID: 34759984 PMC8565636

[13] Gelder CM Hetzel MR . Primary tracheal tumours: a national survey. Thorax. (1993) 48:688–92. doi: 10.1136/thx.48.7.688, PMID: 8153914 PMC464644

[14] Hsu AA Tan EH Takano AM . Lower respiratory tract adenoid cystic carcinoma: its management in the past decades. Clin Oncol (R Coll Radiol). (2015) 27:732–40. doi: 10.1016/j.clon.2015.06.012, PMID: 26160258

[15] Djakovic Z Janevski Z Cesarec V Slobodnjak Z Stancic-Rokotov D . Adenoid cystic carcinoma of distal trachea: A case report. Acta Clin Croat. (2019) 58:777–9. doi: 10.20471/acc.2019.58.04.27, PMID: 32595264 PMC7314305

[16] Hetnal M Kielaszek-Cmiel A Wolanin M Korzeniowski S Brandys P Malecki K . Tracheal cancer: Role of radiation therapy. Rep Pract Oncol Radiother. (2010) 15:113–8. doi: 10.1016/j.rpor.2010.08.005, PMID: 24376936 PMC3863152

[17] Harms W Latz D Becker H Gagel B Herth F Wannenmacher M . Treatment of primary tracheal carcinoma. The role of external and endoluminal radiotherapy. Strahlenther Onkol. (2000) 176:22–7. doi: 10.1007/pl00002300, PMID: 10650832

[18] Carvalho Hde A Figueiredo V Pedreira WL Jr. Aisen S . High dose-rate brachytherapy as a treatment option in primary tracheal tumors. Clinics (Sao Paulo). (2005) 60:299–304. doi: 10.1590/s1807-59322005000400007, PMID: 16138236

[19] Yang Y Ran J Wang Y Zhou Z Chen D Feng Q . Intensity modulated radiation therapy may improve survival for tracheal-bronchial adenoid cystic carcinoma: A retrospective study of 133 cases. Lung Cancer. (2021) 157:116–23. doi: 10.1016/j.lungcan.2021.05.006, PMID: 34020823

[20] Nakamura M Ohnishi K Nakazawa K Shimizu K Miyauchi D Mizumoto M . Long-term follow-up of unresectable adenoid cystic carcinoma of the trachea and bronchus treated with high-dose proton beam therapy: A report of two cases. Thorac Cancer. (2024) 15:201–5. doi: 10.1111/1759-7714.15158, PMID: 37984929 PMC10788470

[21] Schulz-Ertner D Nikoghosyan A Jakel O Haberer T Kraft G Scholz M . Feasibility and toxicity of combined photon and carbon ion radiotherapy for locally advanced adenoid cystic carcinomas. Int J Radiat Oncol Biol Phys. (2003) 56:391–8. doi: 10.1016/s0360-3016(02)04511-x, PMID: 12738314

[22] Jensen AD Nikoghosyan AV Lossner K Haberer T Jakel O Munter MW . COSMIC: A regimen of intensity modulated radiation therapy plus dose-escalated, raster-scanned carbon ion boost for Malignant salivary gland tumors: results of the prospective phase 2 trial. Int J Radiat Oncol Biol Phys. (2015) 93:37–46. doi: 10.1016/j.ijrobp.2015.05.013, PMID: 26279022

[23] Jensen AD Poulakis M Nikoghosyan AV Welzel T Uhl M Federspil PA . High-LET radiotherapy for adenoid cystic carcinoma of the head and neck: 15 years’ experience with raster-scanned carbon ion therapy. Radiother Oncol. (2016) 118:272–80. doi: 10.1016/j.radonc.2015.05.010, PMID: 26164774

[24] Sulaiman NS Demizu Y Koto M Saitoh JI Suefuji H Tsuji H . Multicenter study of carbon-ion radiation therapy for adenoid cystic carcinoma of the head and neck: subanalysis of the Japan carbon-ion radiation oncology study group (J-CROS) study (1402 HN). Int J Radiat Oncol Biol Phys. (2018) 100:639–46. doi: 10.1016/j.ijrobp.2017.11.010, PMID: 29413278

[25] Laurie SA Licitra L . Systemic therapy in the palliative management of advanced salivary gland cancers. J Clin Oncol. (2006) 24:2673–8. doi: 10.1200/JCO.2005.05.3025, PMID: 16763282

