# Supplementary material for: Definitive radiotherapy for adenoid cystic carcinoma of main bronchus: a case report with 10-year follow-up
Source: Front Oncol. 2026 Jan 20;15:1608613. doi: 10.3389/fonc.2025.1608613 (PMC12864126; doi:10.3389/fonc.2025.1608613)
Supplement: Supplementary file 1 [file DataSheet1.docx]

# Supplementary Figure

Supplementary Figure 1. Pre-treatment lung perfusion scan


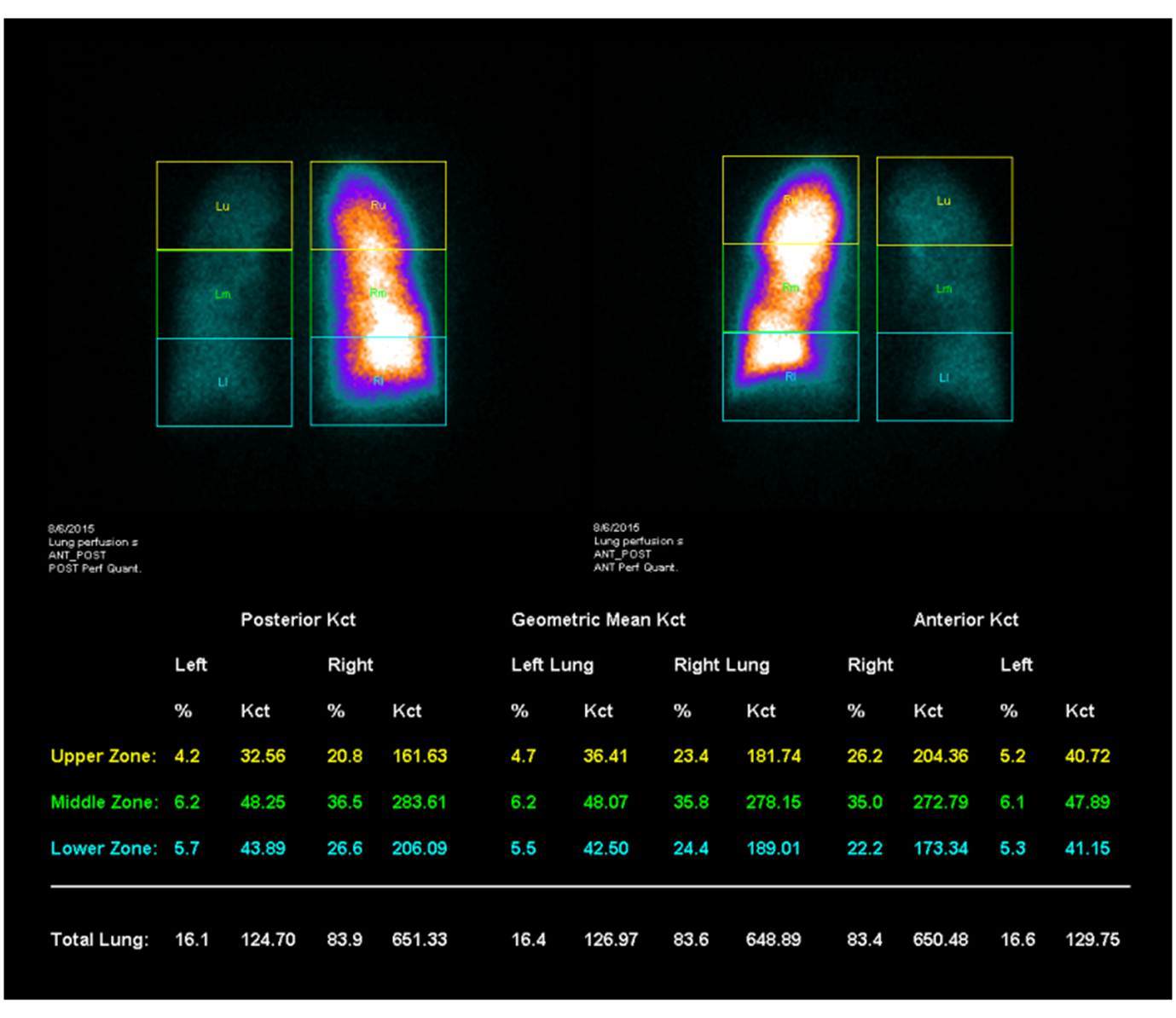


Lung perfusion scan demonstrated markedly reduced perfusion in the left lung (16.4%), with compensatory increased perfusion in the right lung (83.6%). The perfusion defect in the left lung was attributed to narrowing of the left main bronchus, likely caused by the endobronchial mass. Quantitative perfusion analysis revealed significant perfusion reduction across all left lung zones, highlighting the functional impact of bronchial obstruction.

Supplementary Figure 2. (A) Pretreatment positron emission tomography-computed tomography (PET-CT) showed fluorodeoxyglucose (FDG) uptake in the left main bronchial mass. (B) Posttreatment PET-CT showed a significant decrease in FDG uptake, indicating a favorable response to radiotherapy. (C, D) Posttreatment chest CT scans


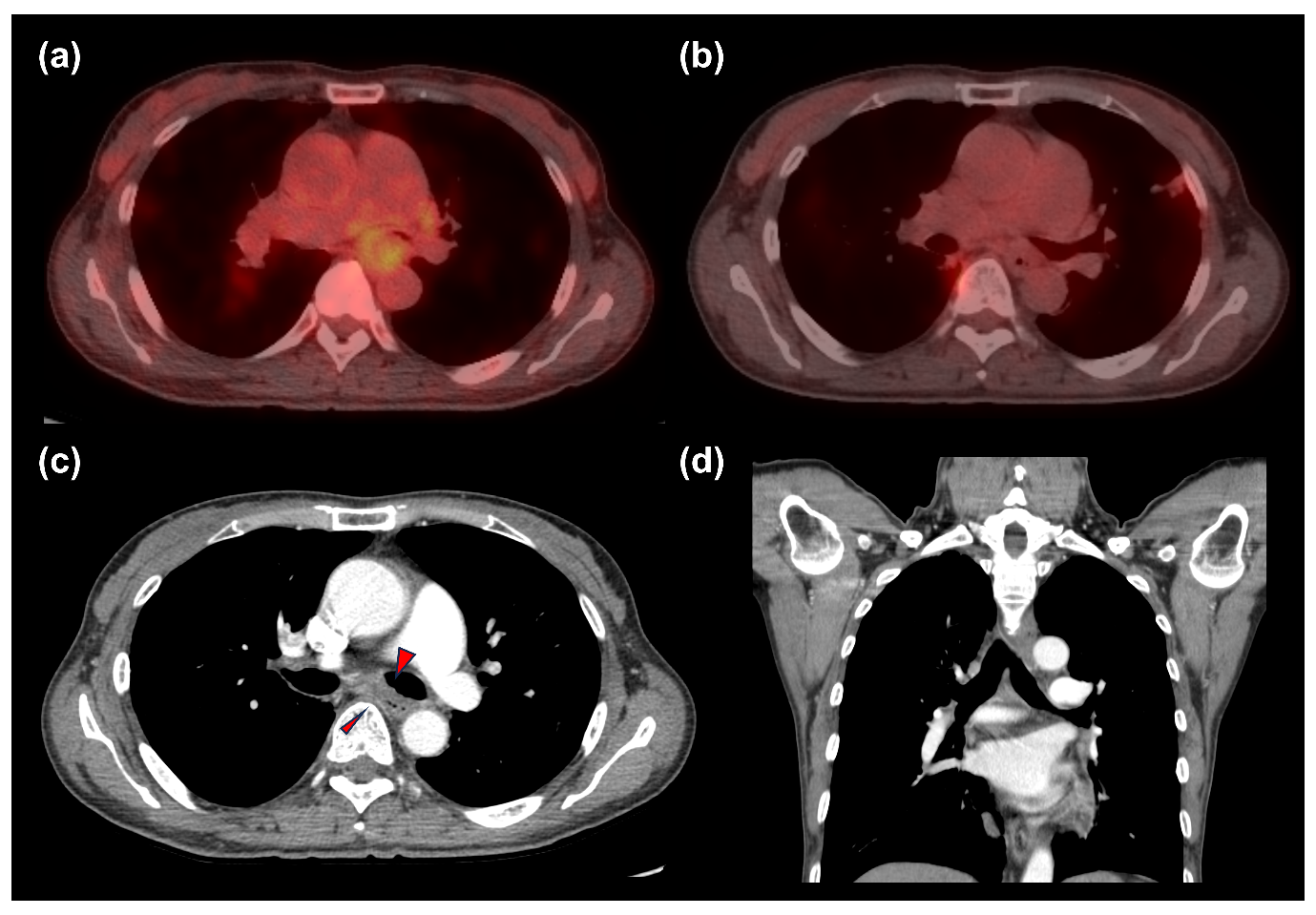


Axial and coronal images showed a marked reduction in bronchial mass size, leaving only minimal residual soft tissue infiltration, consistent with a partial response or better.
